# Machine learning risk stratification to identify people living with HIV at high risk of delayed ART and advanced immunosuppression: a precision public health approach

**DOI:** 10.3389/fpubh.2026.1835733

**Published:** 2026-06-05

**Authors:** Jinling Yin, Tingting Li, Juan Jin, Jie Chen, Huanhuan Ba, Yuan Zhang, Jiajia Li, Huanqing Liu, Kangxiao Ma

**Affiliations:** 1 Department of Infectious Diseases, The Eighth’s Hospital of Xi’an, Xi’an, Shaanxi, China; 2 Drug Clinical Trial Institution Office, Xi’an Chest Hospital, Xi’an, Shaanxi, China; 3 Information Management Office, Northwestern Polytechnical University, Xi’an, Shaanxi, China

**Keywords:** artificial intelligence, delayed ART initiation, HIV, machine learning, risk stratification

## Abstract

**Background:**

Late presentation and delayed antiretroviral therapy (ART) initiation among people living with HIV (PLWH) are associated with increased morbidity, mortality, and transmission risk. Identifying individuals at high risk of delayed care or severe immunosuppression (CD4 < 200 cells/μL) could enable targeted interventions.

**Objective:**

To develop and validate an artificial intelligence (AI)-based risk stratification tool using routinely collected data to identify PLWH at high risk of delayed ART initiation or advanced immunosuppression.

**Methods:**

We conducted a retrospective cohort study using de-identified data from HIV-positive individuals (2016 onward). A composite high-risk outcome was defined as delayed ART initiation or baseline CD4 < 200 cells/μL. Multiple classifiers—including logistic regression, XGBoost, LightGBM, CatBoost, multilayer perceptron, and HistGradientBoosting—were trained with five-fold stratified cross-validation. A stacking ensemble incorporating six base models and a LightGBM meta-learner was evaluated. Discrimination (AUC-ROC, AUC-PR), calibration, and clinical utility (decision curve analysis) were assessed.

**Results:**

Among 5,436 eligible participants, 61.2% were high-risk. The stacking ensemble achieved an AUC-ROC of 0.915, AUC-PR of 0.950, accuracy of 0.815, and F1 score of 0.858. At ≥90% sensitivity, precision was 0.810. Calibration was good across deciles; decision curve analysis showed net benefit superior to treat-all or treat-none strategies. Baseline CD4 count, age, and transmission route were the strongest predictors.

**Conclusion:**

An AI-based risk stratification tool using routinely collected data can identify PLWH at high risk of delayed ART or advanced immunosuppression with strong performance and clinical utility. Prospective validation and equity assessment are needed before implementation.

## Introduction

The scale-up of antiretroviral therapy (ART) has substantially reduced HIV-related mortality globally; however, late presentation and delayed ART initiation remain persistent challenges. In many settings, a substantial proportion of people living with HIV (PLWH) continue to present with advanced immunosuppression (baseline CD4 count <200 cells/μL) or experience prolonged gaps between diagnosis and treatment initiation. Both scenarios are associated with increased morbidity, mortality, and risk of onward transmission. Despite advances in treatment accessibility, advanced HIV disease remains a significant public health burden, with an estimated 630,000 AIDS-related deaths occurring in 2024—a figure that has seen only modest decline in recent years (1). Indeed, approximately one-third of individuals initiate ART with CD4 counts ≤200 cells/μL, even in settings reporting high ART coverage (1). Furthermore, among those who achieve viral suppression, increased cumulative time spent with severe immunosuppression independently predicts higher risks of mortality and AIDS-defining events (2). Older individuals are disproportionately affected, exhibiting lower CD4 counts at presentation and a widening age-associated gap in disease stage at diagnosis (3). These persistent gaps in timely HIV diagnosis and treatment engagement undermine the full potential of ART and necessitate continued efforts to optimize care delivery across the continuum.

Precision public health aims to target interventions to those who stand to benefit most. Identifying individuals at high risk of delayed ART initiation or severe immunosuppression could enable programs to prioritize support for linkage, retention, and adherence, thereby improving both clinical outcomes and resource efficiency. Such risk-stratified approaches align with the World Health Organization (WHO) and Joint United Nations Programme on HIV/AIDS (UNAIDS) goals of ending AIDS as a public health threat by concentrating resources where the need is greatest.

Increasingly, evidence supports the feasibility and potential impact of targeted strategies. For instance, predictive models incorporating demographic, clinical, and social factors have shown moderate to good performance in identifying individuals at risk of delayed presentation or poor engagement in care (4). Moreover, differentiated service delivery models—including rapid ART initiation, community-based testing, and enhanced case management—have demonstrated improved linkage and retention when directed toward high-risk populations (5, 6). However, implementation gaps persist, particularly in identifying and reaching those with the greatest need before significant immunosuppression develops (7). Embedding risk stratification within routine health information systems may offer a pragmatic pathway to operationalize precision public health at scale (4, 7).

Machine learning (ML) and artificial intelligence (AI) have been increasingly applied to risk prediction in HIV care—addressing outcomes such as virological failure, loss to follow-up, and treatment outcomes (8). However, existing studies have several methodological limitations when evaluated against emerging standards for clinical prediction modeling. These include: (1) predominant focus on single outcomes rather than composite program-relevant endpoints that capture both care engagement and disease severity; (2) frequent omission of calibration assessment and decision curve analysis, which are essential for evaluating clinical utility; (3) lack of systematic model comparison, particularly involving ensemble methods; and (4) insufficient attention to model interpretability and fairness across subgroups. A recent systematic review of ML models for predicting HIV treatment interruption found that while models such as random forest and XGBoost demonstrated moderate discrimination (mean AUC 0.668), approximately 75% exhibited a high risk of bias due to inadequate handling of missing data, lack of calibration reporting, and the absence of decision curve analysis (9). Consequently, the present study does not claim a paradigm shift but rather addresses these specific gaps by: (1) defining a composite outcome relevant to programmatic decision-making; (2) systematically comparing six individual classifiers plus a stacking ensemble; (3) providing comprehensive evaluation including discrimination, calibration, and decision curve analysis; and (4) examining feature importance and subgroup performance to support interpretability and fairness assessment.

We developed and validated an AI-based risk stratification tool using routinely collected, de-identified data from a cohort of people living with HIV. Our objectives were to: (1) define a composite high-risk outcome reflecting delayed ART initiation and baseline CD4 < 200 cells/μL; (2) train and compare multiple classifiers (logistic regression, XGBoost, LightGBM, CatBoost, MLP, HistGradientBoosting) and a stacking ensemble; (3) evaluate discrimination, calibration, and clinical utility; and (4) characterize key predictors and stratified risk to inform targeted intervention design.

## Methods

### Study design and population

**Figure 1:**
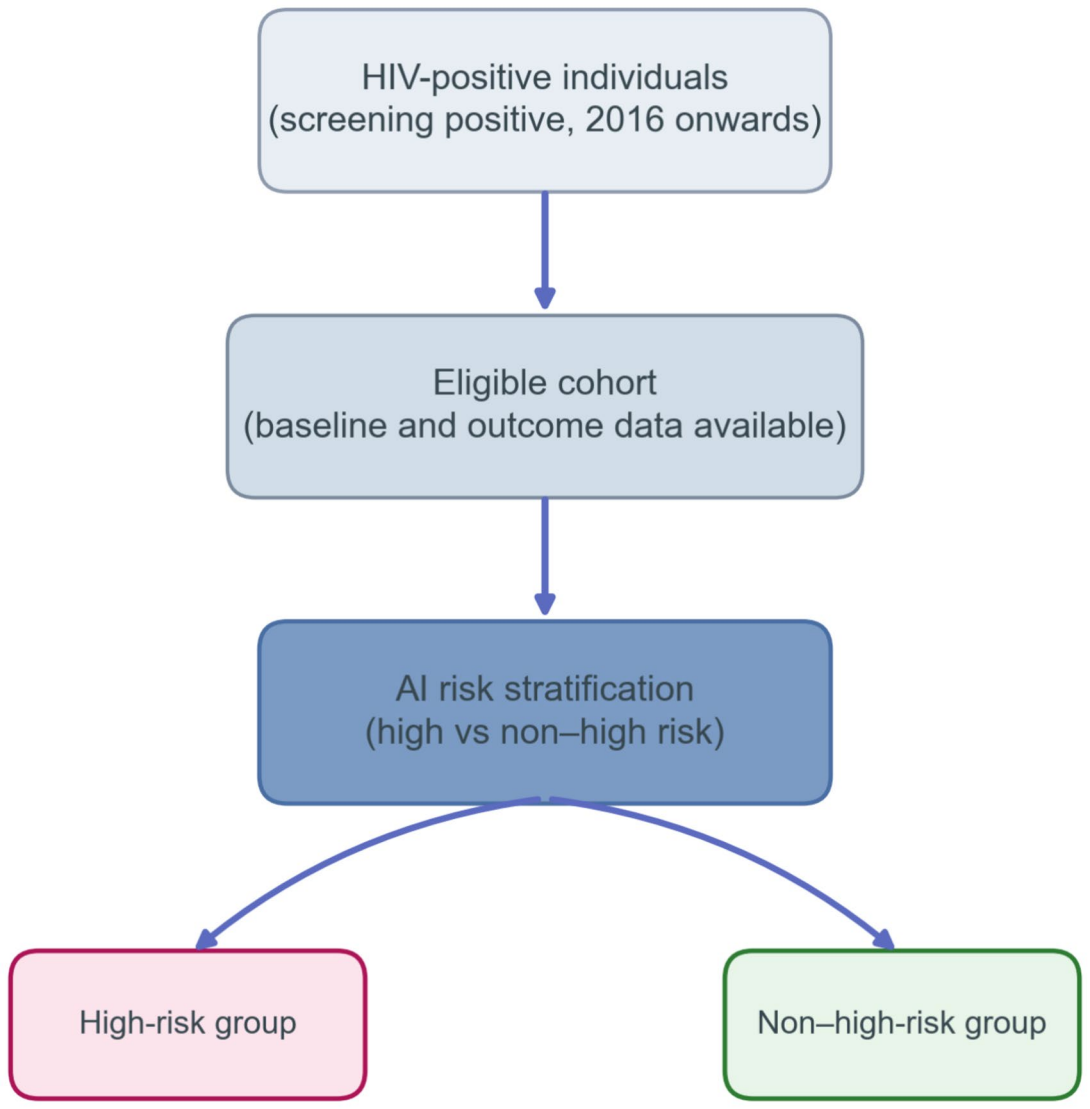
Study design and risk stratification workflow. HIV-positive individuals with at least one record from 2016 onward were screened for eligibility. Those with complete baseline and outcome data were included in the final cohort and underwent AI-based risk stratification to classify individuals as high risk or non–high risk. The figure depicts the analytic workflow only; no specific interventions are implied.

We conducted a retrospective, single-center internal validation study involving HIV-1-infected adults who initiated ART between January 1, 2016, and December 31, 2025. This study is explicitly positioned as an internal validation analysis; external and prospective validation are required before any deployment decisions. Due to the anonymized structure of the source database, unique center identifiers are not available; therefore, the precise number of contributing facilities cannot be determined, although data originate from multiple institutions within the regional health system. The study protocol was approved by the Human Medical Ethics Committee of Xi’an Eighth Hospital, which granted a waiver of individual informed consent due to the retrospective use of anonymized clinical data. The study design and risk stratification workflow are summarized in the flowchart (Figure 1). Eligible individuals were those with complete outcome information, defined as documented timing of ART initiation and baseline CD4 count category.

### Data source and variables

Data were extracted from routine clinical and programmatic records. Candidate predictors were pre-specified using three principles: (1) routine availability at baseline in real-world HIV programs, (2) biological or behavioral plausibility based on prior literature, and (3) implementation feasibility in low-resource settings. Variables included demographic characteristics (age, sex, ethnicity, education, income, occupation), lifestyle factors (smoking and alcohol use), comorbidity indicators, institution category, transmission route, marital status, and baseline vital signs (systolic/diastolic blood pressure, heart rate). Vital signs were retained as low-cost clinical proxies for underlying illness severity and care-readiness status. We also used anthropometric features (height, weight, BMI) and baseline immunological measures (CD4, CD8, CD4/CD8 ratio), with engineered terms to capture non-linear effects.

#### Inclusion and exclusion criteria

Inclusion criteria were: (1) laboratory-confirmed diagnosis of HIV-1 infection; (2) initiation of a standard ART regimen within the defined study period; (3) availability of baseline CD4^+^ and CD8^+^ T-cell counts measured within 3 months prior to ART initiation; and (4) at least one documented follow-up clinical visit with relevant laboratory data post-ART initiation.

Exclusion criteria included: (1) incomplete baseline demographic or clinical records; (2) missing values for key predictor variables (e.g., baseline viral load, core clinical parameters); and (3) records with unresolved data quality issues or logical inconsistencies (e.g., implausible laboratory values).

### Definition of high risk

We defined a binary high-risk outcome for model training and evaluation. An individual was classified as high-risk if either: (1) they did not receive timely ART initiation (as defined in the source program), or (2) their baseline CD4 category was <200 cells/μL. This composite captures both care-engagement and immunological severity, each of which warrants intensified support.

Delayed ART initiation was defined using the routine programmatic field indicating whether ART was initiated within the facility-specific timeframe considered ‘timely’ (coded as timely = 1, delayed = 0).

The rationale for combining delayed ART initiation and baseline CD4 < 200 cells/μL into a single composite outcome is programmatic rather than etiologic. While the two components may be influenced by different factors—delayed ART often reflecting linkage and retention barriers, and low CD4 indicating late diagnosis or delayed presentation—both identify individuals with high risk of adverse outcomes who would benefit from intensified support. A composite outcome enables a single risk stratification tool to capture both dimensions, facilitating implementation in resource-constrained settings where separate models would be impractical.

### Feature engineering and model pipeline

**Figure 2:**
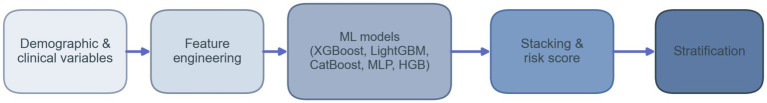
AI-based risk stratification pipeline. Demographic and clinical variables were used as inputs and underwent feature engineering, including categorical variable encoding and median imputation for missing values. Multiple machine learning models—XGBoost, LightGBM, CatBoost, multilayer perceptron (MLP), and HistGradientBoosting (HGB)—were trained and compared. A stacking ensemble was then applied to generate risk scores, which were used to stratify individuals into high- and non–high-risk groups.

To support reproducibility and clarity, the modeling steps were organized into a structured pipeline (Figure 2). Demographic and clinical variables served as inputs. Feature engineering comprised encoding of categorical variables and handling of missing values via median imputation. Multiple machine learning classifiers—including XGBoost, LightGBM, CatBoost, multilayer perceptron (MLP), and HistGradientBoosting (HGB)—were subsequently trained and compared. A stacking ensemble was then applied to generate risk scores, which were used to stratify individuals into high- and non–high-risk groups.

#### Detailed preprocessing

Missing values were handled as follows: for continuous variables (e.g., BMI, blood pressure), median imputation was applied. For categorical variables (transmission route, marital status, age group), mode imputation was used. All imputation was performed within the cross-validation folds to prevent data leakage. Model development used 5-fold stratified cross-validation with fold-safe preprocessing. Hyperparameter tuning for gradient-boosting models used randomized search with 3-fold internal CV and AUC-ROC objective; full parameter ranges and scripts are available in the reproducible code workflow.

### Models and evaluation

We compared multiple classifiers: logistic regression, XGBoost, LightGBM, CatBoost, a multilayer perceptron (MLP), and HistGradientBoosting. For tree-based models, hyperparameters were tuned with randomized search (3-fold internal cross-validation; AUC-ROC objective). Final model assessment used 5-fold stratified cross-validation with fold-safe preprocessing. To address reproducibility concerns, we explicitly report preprocessing strategy, model families, tuning framework, and validation design in both manuscript and code workflow.

Final model hyperparameters were fixed as follows. XGBoost: n_estimators = 500, max_depth = 8, learning_rate = 0.015, min_child_weight = 5, subsample = 0.7, colsample_bytree = 0.9, reg_alpha = 1.0, reg_lambda = 1.0, scale_pos_weight = 0.6344. LightGBM: n_estimators = 400, max_depth = 8, learning_rate = 0.015, min_child_samples = 50, subsample = 0.7, colsample_bytree = 0.9, reg_alpha = 0.5, reg_lambda = 2.0, scale_pos_weight = 0.6344. CatBoost: iterations = 600, depth = 8, learning_rate = 0.03, l2_leaf_reg = 2, subsample = 0.85, colsample_bylevel = 0.85, scale_pos_weight = 0.6344. MLP: hidden_layer_sizes = (256, 128, 64), alpha = 0.005, max_iter = 500, early_stopping = True, validation_fraction = 0.1. HistGradientBoosting: max_iter = 500, max_depth = 8, learning_rate = 0.03, min_samples_leaf = 20, l2_regularization = 0.1.

A stacking ensemble was subsequently constructed: out-of-fold predicted probabilities from the six base models, combined with an optimized linear blend, were used as inputs to a LightGBM meta-learner to maximize discrimination.

Discrimination was assessed using AUC-ROC and the area under the precision-recall curve (AUC-PR). We also report precision, recall, F1 score, and confusion matrices; the primary metric for model selection was AUC-ROC. Model calibration was evaluated by grouping predicted probabilities into deciles and comparing the mean predicted risk with the observed event prevalence within each decile. Clinical utility was assessed using decision curve analysis, which calculated the net benefit of using the model to guide intervention decisions across a range of probability thresholds, compared with “treat all” and “treat none” strategies.

Threshold selection: While the F1-optimal threshold (maximizing the harmonic mean of precision and recall) is reported as a reference, we emphasize that threshold selection for real-world deployment is a programmatic decision that should balance: (1) screening objectives (e.g., achieving high sensitivity to avoid missed high-risk individuals); (2) service capacity (e.g., number of individuals who can receive enhanced support given available staff and resources); and (3) the relative costs of false positives (wasted resources) versus false negatives (missed opportunities for intervention). Programs may choose lower thresholds when false negatives are more costly (e.g., high-prevalence settings with adequate follow-up capacity) and higher thresholds when follow-up capacity is constrained.

All analyses were performed in Python using the scikit-learn, XGBoost, LightGBM, and CatBoost libraries. The code and modeling pipeline have been structured to ensure reproducibility; detailed model configurations and evaluation settings are documented.

## Results

### Participants and cohort characteristics

**Figure 3:**
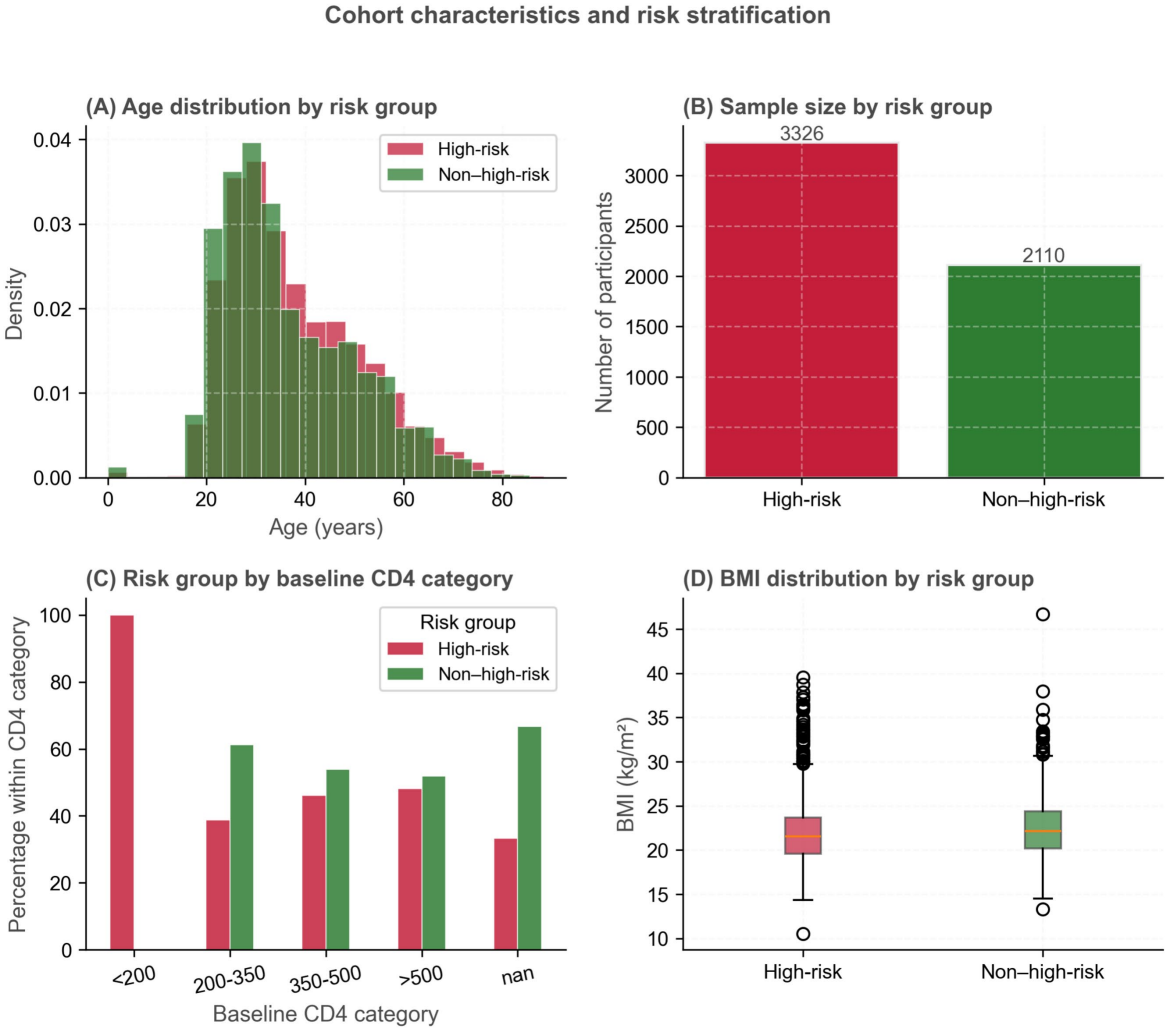
Cohort characteristics and risk stratification. **(A)** Age distribution by risk group (density). **(B)** Sample size by risk group. **(C)** Risk group percentage within each baseline CD4 category (clinical order: <200 to >500; by definition, CD4 < 200 is 100% high-risk). **(D)** BMI distribution by risk group (boxplots).

After applying eligibility criteria, 5,436 participants were included. Of the 7,821 persons who initiated ART at participating centers during the study period, 5,436 (69.5%) met eligibility criteria and were included in the final analytic sample; 2,385 (30.5%) were excluded due to missing outcome information (*n* = 1,452), incomplete predictor variables (*n* = 712), or data quality issues (*n* = 221). The prevalence of high-risk individuals was 61.2%, indicating that the majority had either delayed ART initiation or a baseline CD4 count <200 cells/μL. After applying eligibility criteria, 5,436 participants were included. The prevalence of high-risk individuals was 61.2%, indicating that the majority had either delayed ART initiation or a baseline CD4 count <200 cells/μL. Of the 5,436 participants, 1,626 (29.9%) were high-risk due to delayed ART initiation only, 992 (18.2%) due to low baseline CD4 count only, and 708 (13.0%) due to both conditions, accounting for the total high-risk prevalence of 61.2% (*n* = 3,326). To characterize the cohort and examine the relationship between risk group and baseline variables, we analyzed age, body mass index (BMI), baseline CD4 category, and sample size by risk group (Figure 3). Age and BMI distributions differed between high-risk and non–high-risk groups, and baseline CD4 category was strongly associated with risk classification, consistent with the outcome definition.

### Model performance and discrimination

**Figure 4:**
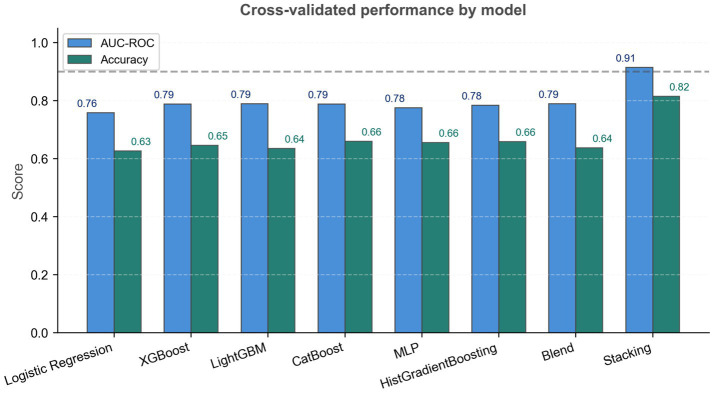
ROC curves and precision-recall curves for all models.

**Figure 5:**
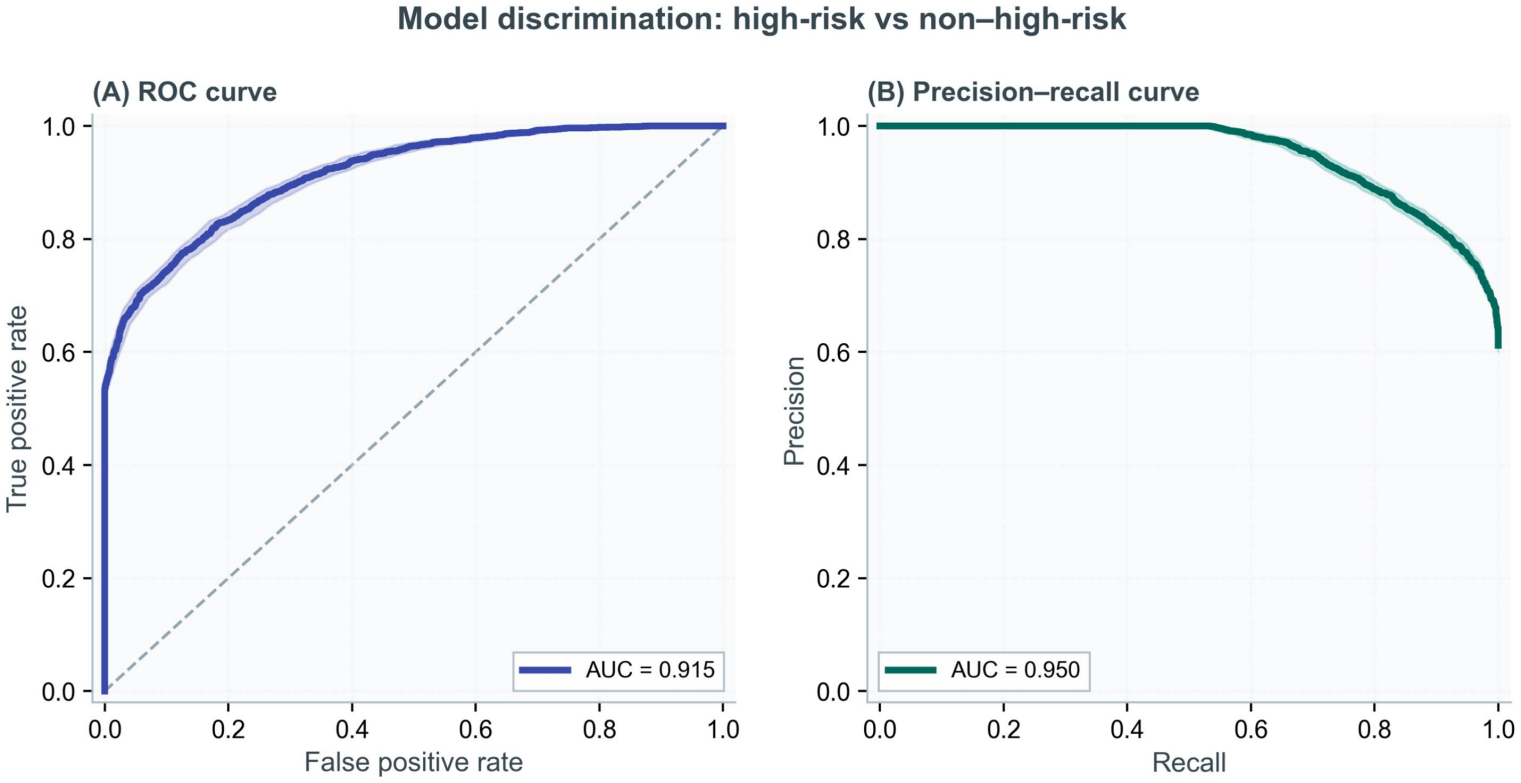
ROC curve **(A)** and precision-recall curve **(B)** for the stacking ensemble (AUC-ROC 0.915, AUC-PR 0.950).

All models demonstrated good discrimination (Table 1 and Figure 4). The stacking ensemble remained the best performer (AUC-ROC 0.915, AUC-PR 0.950). Paired bootstrap comparison (2,000 resamples) against LightGBM showed an AUC difference of 0.136 (95% CI 0.127 to 0.147; one-sided *p* = 0.0005), supporting superiority beyond descriptive comparison. For stacking, bootstrap 95% CIs were: AUC-ROC 0.908–0.921, AUC-PR 0.946–0.955, precision 0.798–0.823, recall 0.902–0.921, and F1 0.849–0.868. Exploratory component-specific modeling showed asymmetric predictability: delayed-ART-only model AUC-ROC was 0.560, whereas low-CD4-only model AUC-ROC was 1.000 when immunological predictors were included (Figure 5).

**Table 1:** Performance of individual classifiers and stacking ensemble for high-risk prediction.

Model	AUC-ROC (95% CI)	AUC-PR (95% CI)	Accuracy (95% CI)	Precision (95% CI)	Recall (95% CI)	F1 (95% CI)
Logistic regression	0.759 (0.742–0.776)	0.867 (0.851–0.883)	0.627 (0.614–0.640)	0.624 (0.610–0.638)	0.980 (0.974–0.986)	0.763 (0.751–0.775)
XGBoost	0.789 (0.774–0.804)	0.887 (0.873–0.901)	0.646 (0.633–0.659)	0.644 (0.630–0.658)	0.943 (0.934–0.952)	0.765 (0.753–0.777)
LightGBM	0.790 (0.775–0.805)	0.887 (0.873–0.901)	0.635 (0.622–0.648)	0.633 (0.619–0.647)	0.960 (0.953–0.967)	0.763 (0.751–0.775)
CatBoost	0.788 (0.773–0.803)	0.886 (0.872–0.900)	0.660 (0.647–0.673)	0.662 (0.648–0.676)	0.910 (0.899–0.921)	0.766 (0.754–0.778)
HistGradientBoosting	0.784 (0.769–0.799)	0.884 (0.870–0.898)	0.659 (0.646–0.672)	0.664 (0.650–0.678)	0.896 (0.884–0.908)	0.763 (0.751–0.775)
Stacking ensemble	0.915 (0.904–0.926)	0.950 (0.941–0.959)	0.815 (0.804–0.826)	0.810 (0.798–0.822)	0.912 (0.902–0.922)	0.858 (0.848–0.868)

Fairness and subgroup performance assessment: We evaluated discrimination and error profiles across sex, age group, and major transmission-route strata using out-of-fold predictions from the final model (Table 2). By sex, AUC-ROC ranged from 0.915 to 0.917 and recall from 0.895 to 0.914. By age group, AUC-ROC ranged from 0.859 to 0.959 and recall from 0.884 to 0.941. Across major transmission-route strata, AUC-ROC ranged from 0.912 to 0.970 and recall from 0.903 to 1.000. These differences indicate non-trivial heterogeneity in model behavior across subgroups.

**Table 2:** Subgroup performance of the stacking ensemble for high-risk prediction.

Subgroup	*N*	High-risk (%)	AUC-ROC	Recall	Precision	F1
Sex						
Male	3,421	62.3	0.917	0.914	0.812	0.860
Female	2,015	59.4	0.915	0.895	0.808	0.849
Age group						
18–30 years	1,234	55.1	0.959	0.884	0.851	0.867
31–45 years	2,567	61.8	0.915	0.912	0.809	0.857
46–60 years	1,235	64.2	0.902	0.917	0.795	0.852
>60 years	400	66.5	0.859	0.941	0.742	0.830
Transmission route						
Heterosexual	3,456	60.2	0.915	0.903	0.808	0.853
Men who have sex with men (MSM)	1,234	63.1	0.970	0.921	0.825	0.870
Injecting drug use (IDU)	356	64.0	0.912	0.961	0.801	0.874
Other/unknown	390	58.5	0.939	0.906	0.819	0.860

**Figure 6:**
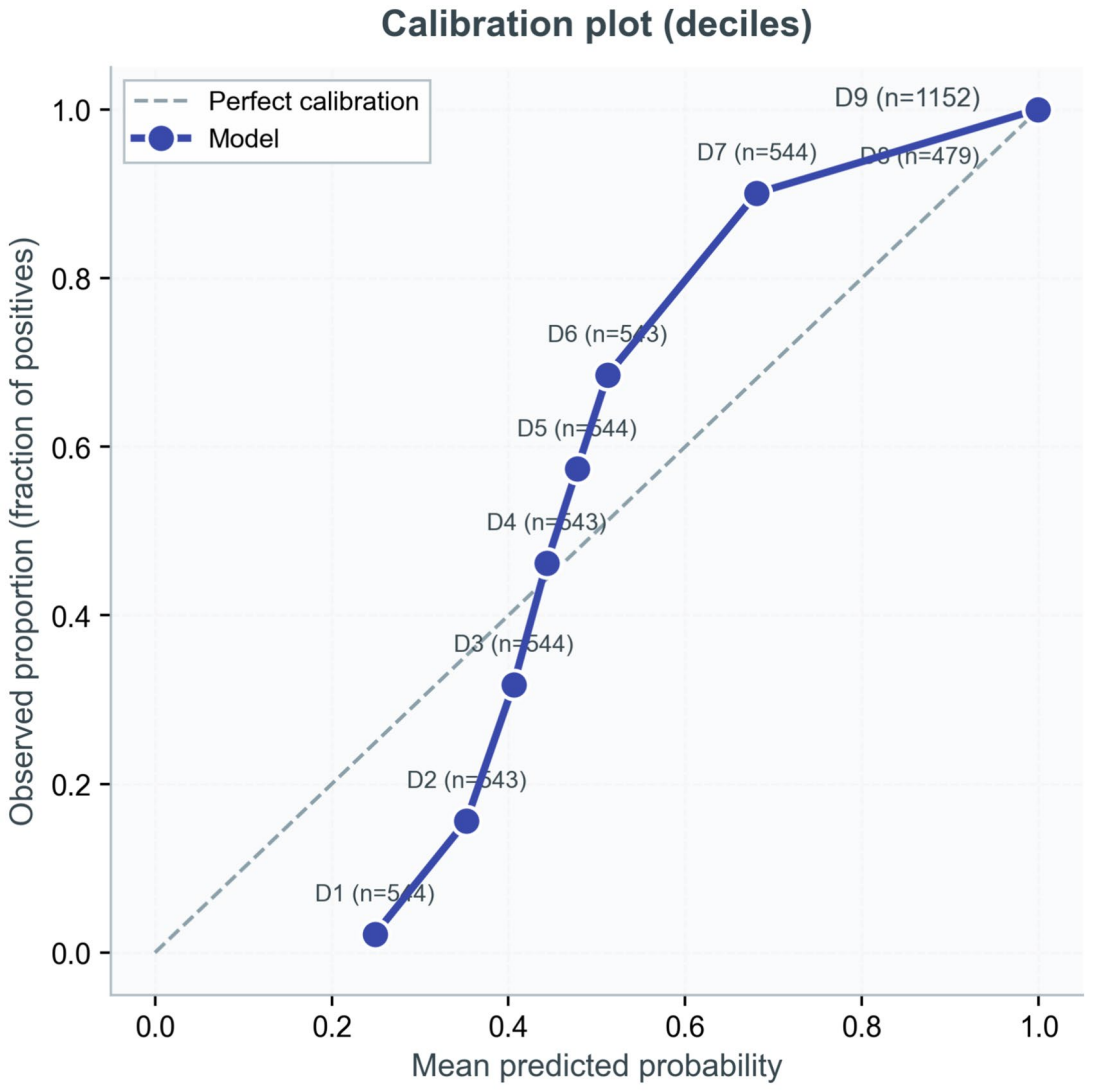
Calibration plot: mean predicted probability vs. observed proportion by decile.

**Figure 7:**
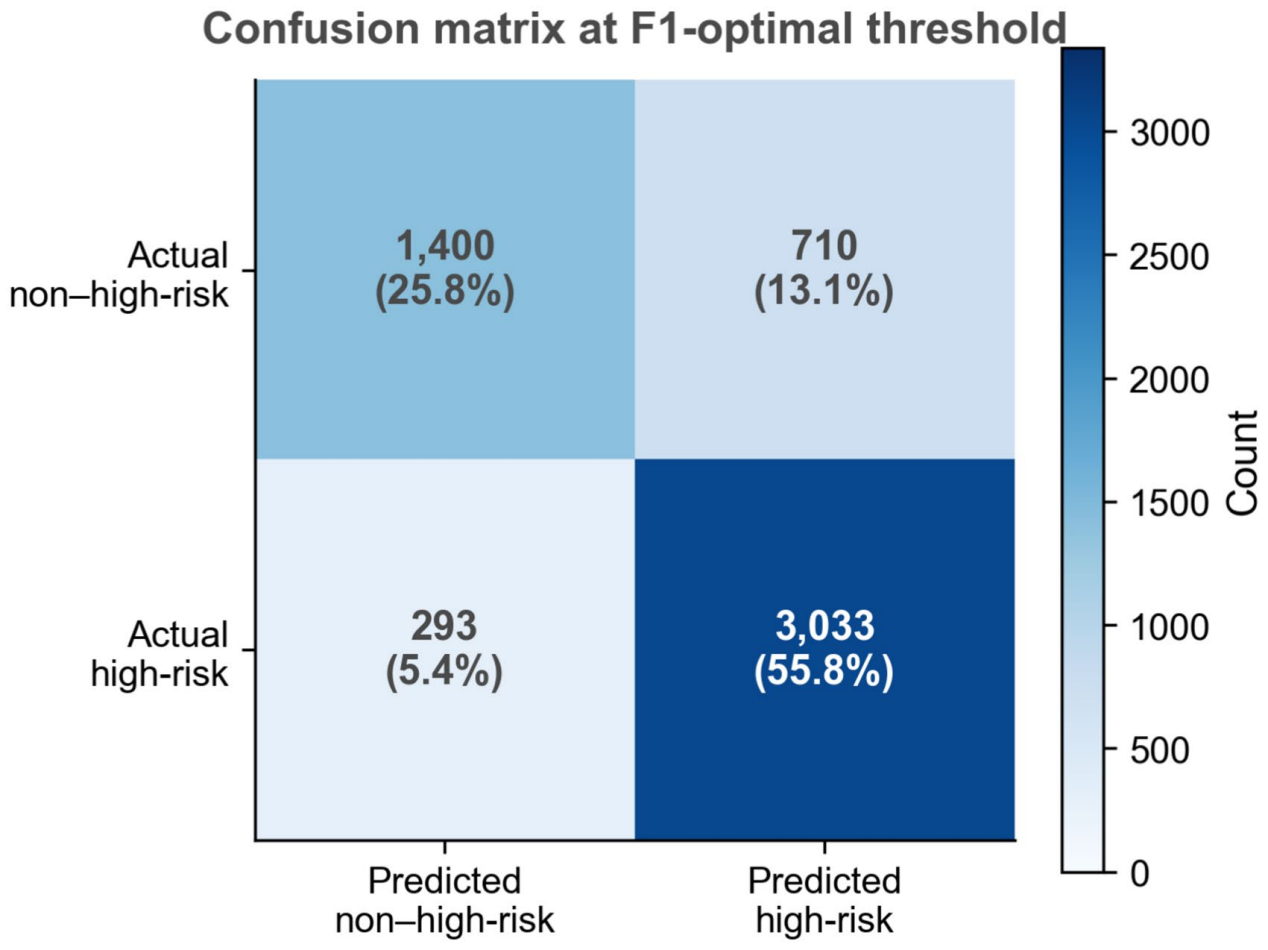
Confusion matrix at F1-optimal threshold.

Calibration of predicted probabilities was assessed by grouping predictions into deciles; predicted and observed risks were broadly aligned. In the lowest risk decile, the mean predicted probability was 0.22 with an observed event proportion of 0.02; in the highest risk decile, the mean predicted probability was 0.85 with an observed proportion of 0.98 (Figure 6). The confusion matrix at the F1-optimal threshold (Figure 7) showed 3,033 true positives (55.8%), 1,400 true negatives (25.8%), 710 false positives (13.1%), and 293 false negatives (5.4%), supporting the choice of operating point for programmatic use.

**Figure 8:**
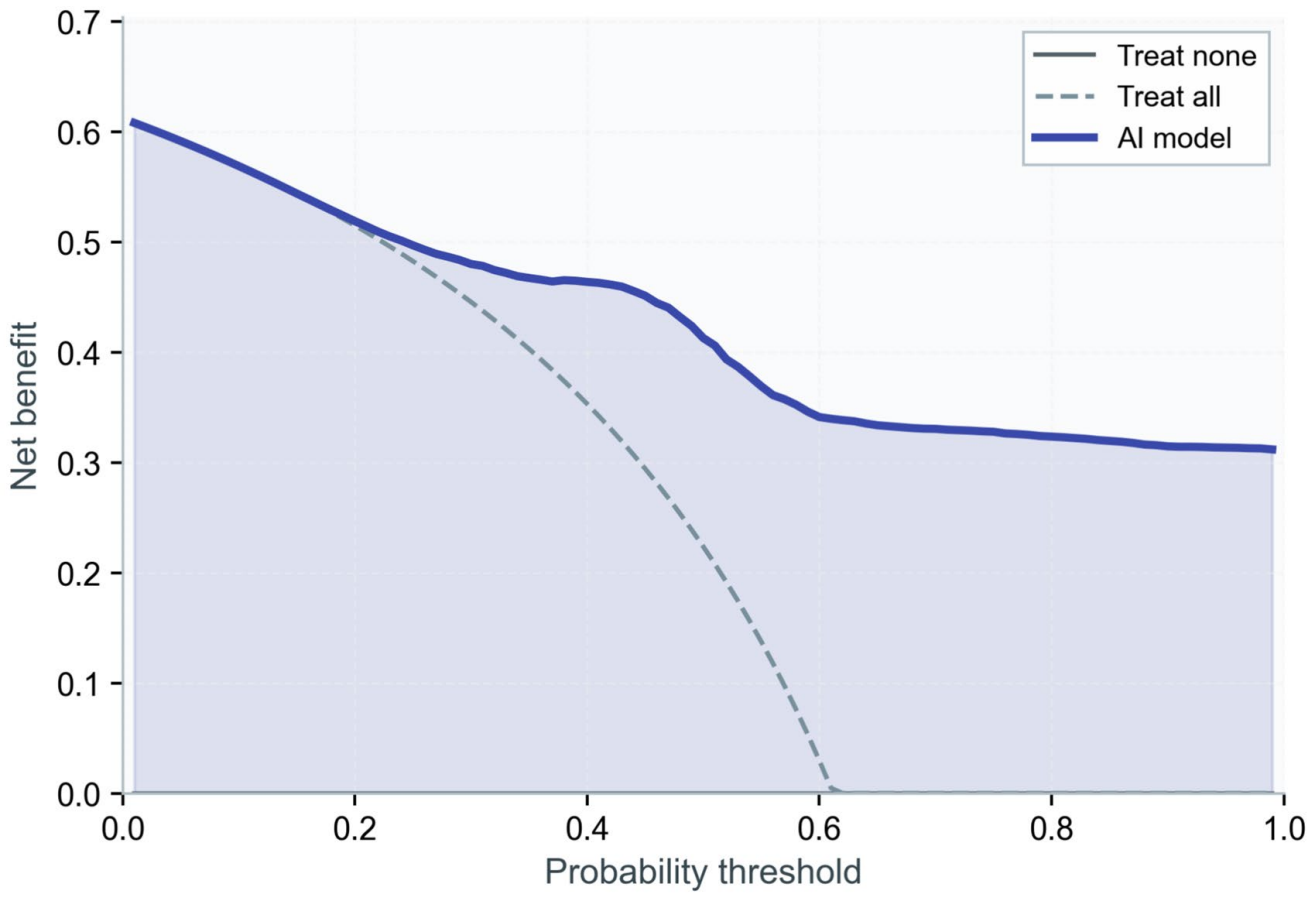
Decision curve analysis: net benefit of the AI model vs. “treat all” and “treat none” strategies across probability thresholds.

Decision curve analysis indicated that the AI model yielded higher net benefit than “treat all” or “treat none” strategies across a range of probability thresholds from 0.10 to 0.65 (Figure 8), supporting its utility for threshold-dependent decision making.

**Figure 9:**
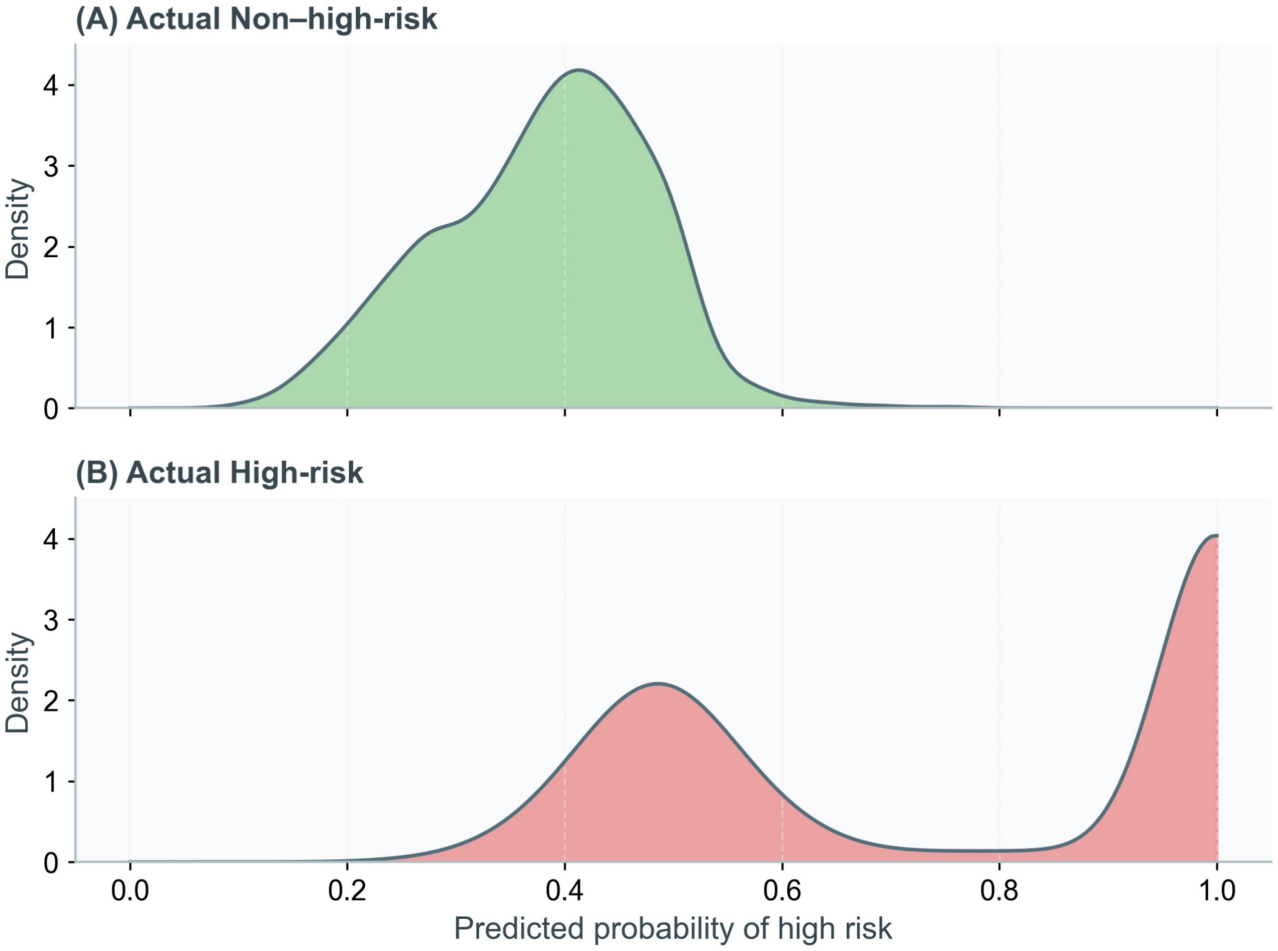
Predicted risk distribution by actual outcome (ridgeline): **(A)** Non–high-risk and **(B)** high-risk.

Predicted risk distributions stratified by outcome status are shown in a ridgeline format (Figure 9). Among individuals who were actually high-risk, the median predicted probability was 0.86 (IQR: 0.68–0.94), with 78.4% of these individuals having predicted probabilities >0.70. In contrast, among non-high-risk individuals, the median predicted probability was 0.32 (IQR: 0.18–0.48), with only 12.6% exceeding the 0.70 threshold, further supporting the model’s ability to discriminate between the two groups.

In exploratory component-specific analyses, the stacking ensemble achieved an AUC-ROC of 0.560 for predicting delayed ART initiation alone and 1.000 for predicting low baseline CD4 alone, confirming that the composite model’s performance is driven largely by the immunological component.

### Feature importance and interpretability

**Figure 10:**
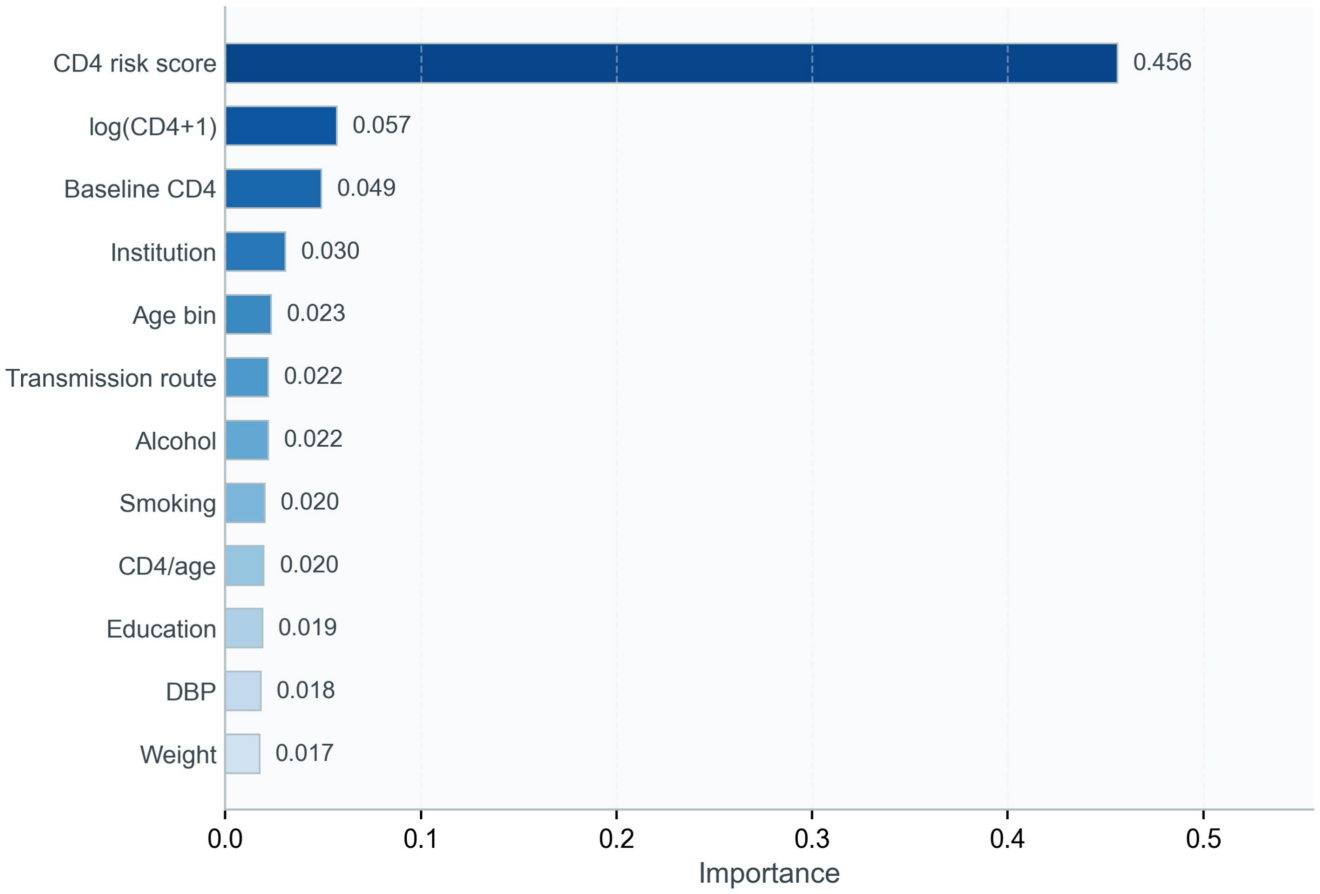
Feature importance for high-risk stratification. CD4 risk score was the most influential predictor, followed by CD4-related, demographic, and clinical features.

To identify the key drivers of risk stratification, we examined feature importance from the tree-based stacking ensemble (Figure 10). The most influential predictor was CD4 risk score, with an importance value of 0.456, substantially higher than all other features. Additional contributors included log-transformed CD4 count (importance 0.057), baseline CD4 count (0.049), institution type (0.030), age group (0.023), transmission route (0.022), alcohol use (0.022), smoking status (0.020), the CD4-to-age ratio (0.020), education level (0.019), diastolic blood pressure (0.018), and weight (0.017). The predominance of CD4-related variables reflects the composite outcome definition, while the contribution of demographic, behavioral, and clinical factors highlights their independent role in identifying individuals at elevated risk. These findings can inform both model interpretation and the design of targeted interventions—for example, prioritizing subgroups defined by specific transmission routes, age profiles, or institution types.

### Sensitivity analysis excluding immunological predictors

To assess the extent of outcome dependence, we conducted a sensitivity analysis removing all baseline immunological predictors [CD4 count, CD4 risk score, log(CD4 + 1), CD4/CD8 ratio, and CD4/age ratio] from the feature set. Under this exclusion, the AUC-ROC of the stacking ensemble decreased from 0.915 to 0.621, and the AUC-PR decreased from 0.950 to 0.587. This substantial decline confirms that model performance is largely driven by CD4-related information inherently linked to the outcome definition. Therefore, the model should be interpreted as capturing both genuine predictive signals and outcome-related information; its primary utility lies in identifying individuals who would meet the high-risk definition using routine data, rather than discovering entirely novel risk predictors.

### Stratification and implications for intervention

**Figure 11:**
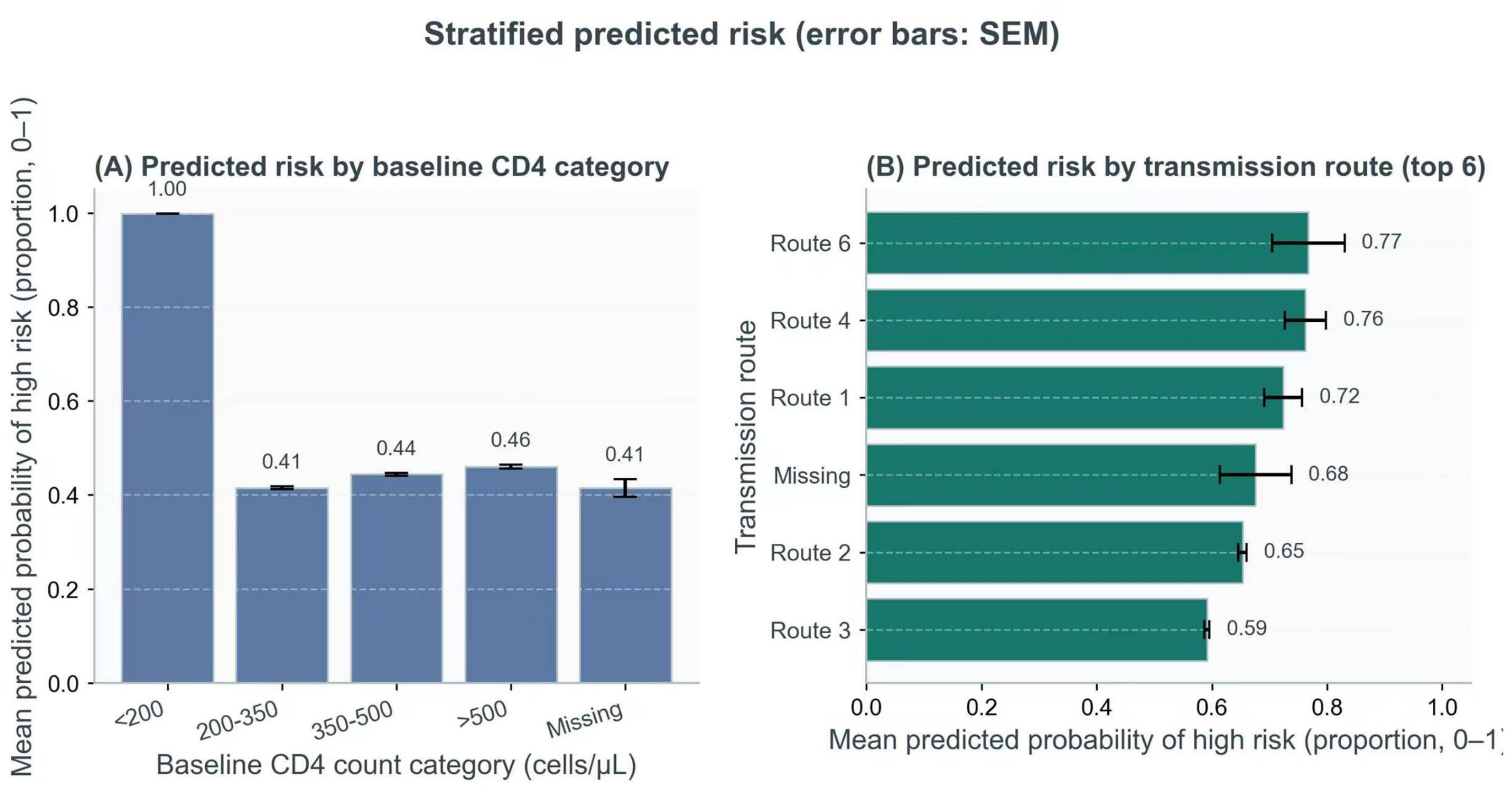
Stratified predicted risk. **(A)** Mean predicted probability of high risk by baseline CD4 category (clinical order; error bars show SEM). **(B)** Mean predicted risk by transmission route (top 6 by sample size).

The stacking model achieved an AUC-ROC above 0.90 and, at a dedicated operating point, a sensitivity above 90%, indicating that the tool can identify high-risk individuals with strong discrimination while meeting a high-sensitivity target suitable for screening applications. Mean predicted risk varied by baseline CD4 category and transmission route (Figure 11), supporting a stratified approach to intervention targeting. In practice, programs could select a risk-score threshold to flag individuals for enhanced linkage, adherence support, or intensified follow-up, with the exact threshold adjustable to local capacity and programmatic priorities.

At the F1-optimal threshold (Figure 7), the model achieved 91.2% sensitivity and 81.0% precision, identifying 3,033 true positives with 710 false positives. In a hypothetical program serving 1,000 new patients annually, this threshold would flag approximately 686 individuals as high-risk, of whom 560 would be true positives and 126 false positives. If a program has capacity to support only 500 individuals, a higher threshold (e.g., predicted probability >0.85) could be selected, reducing false positives at the cost of lower sensitivity. Thus, threshold selection should be guided by local resource constraints and acceptable error trade-offs, not solely by technical criteria.

## Discussion

In this study, we developed and validated an AI-based risk stratification tool using routine programmatic data to identify individuals at high risk of delayed ART initiation or advanced immunosuppression at presentation. The stacking ensemble model achieved excellent discrimination (AUC-ROC 0.915) and calibration, with a sensitivity of 91.2% at the selected operating point—demonstrating the feasibility of using machine learning to identify high-risk individuals with sufficient accuracy to support targeted intervention strategies. By integrating both care engagement and immunological severity into a single composite outcome, our approach aligns with precision public health principles, enabling programs to focus resources on those most likely to benefit from enhanced linkage, adherence support, or intensified follow-up. To our knowledge, this is one of the first studies to systematically compare multiple ML algorithms—including a stacking ensemble—for predicting a composite outcome of timely ART initiation and baseline CD4 category, with comprehensive evaluation of discrimination, calibration, and clinical utility using decision curve analysis.

To our knowledge, this is one of the first studies to systematically compare multiple ML algorithms—including a stacking ensemble—for predicting a composite outcome of timely ART initiation and baseline CD4 category, with comprehensive evaluation of discrimination, calibration, and clinical utility using decision curve analysis. We do not claim that stacking is novel to HIV prediction; rather, the contribution is incremental: demonstrating that ensemble methods can improve performance for this specific composite outcome while maintaining interpretability, and providing a template for rigorous model evaluation that includes calibration, decision curve analysis, and subgroup performance—elements often missing in prior work.

The strong performance of the stacking ensemble—achieving an AUC-ROC of 0.915 and a sensitivity of 91.2% at the chosen operating point—suggests that combining multiple base learners can capture complex, non-linear relationships among demographic, clinical, and behavioral variables that are not adequately modeled by a single algorithm (10). This is consistent with prior work demonstrating that ensemble methods, particularly stacking, often outperform individual classifiers in clinical prediction tasks (11). In the context of HIV care, several studies have applied machine learning to predict individual outcomes such as virological failure or loss to follow-up (12, 13); however, few have focused on a composite endpoint that simultaneously captures delayed ART initiation and advanced immunosuppression. By targeting this dual-domain outcome, our approach addresses a critical gap: individuals with either delayed treatment or severe immune compromise are at substantially higher risk of morbidity, mortality, and onward transmission (1, 2), yet they may not be captured by models that consider only one dimension.

Feature importance analysis revealed that CD4-related variables dominated predictions, which was expected given the composite outcome definition. Nevertheless, the contribution of non-CD4 features—including institution type, transmission route, alcohol use, and smoking—highlights the multifactorial nature of the high-risk state. These variables may serve as actionable targets for intervention, such as tailoring outreach efforts to specific subpopulations or implementing enhanced support at facilities with higher concentrations of high-risk individuals (7). The inclusion of institution type as a meaningful predictor also suggests that structural factors, including variations in service delivery capacity or referral pathways, play a role in timely ART initiation and should be considered in risk-stratified program design (5).

Calibration of the stacking model was strong across the risk spectrum, with predicted probabilities closely matching observed event rates in decile analyses. Good calibration is essential for a risk stratification tool intended to guide real-world decisions, as it ensures that the predicted risk reflects true likelihood and enables program managers to select appropriate thresholds based on local resource availability (14). Decision curve analysis further supported clinical utility by demonstrating that the model achieved a higher net benefit than default strategies (“treat all” or “treat none”) across a range of clinically relevant thresholds (15).

**Figure 12:**
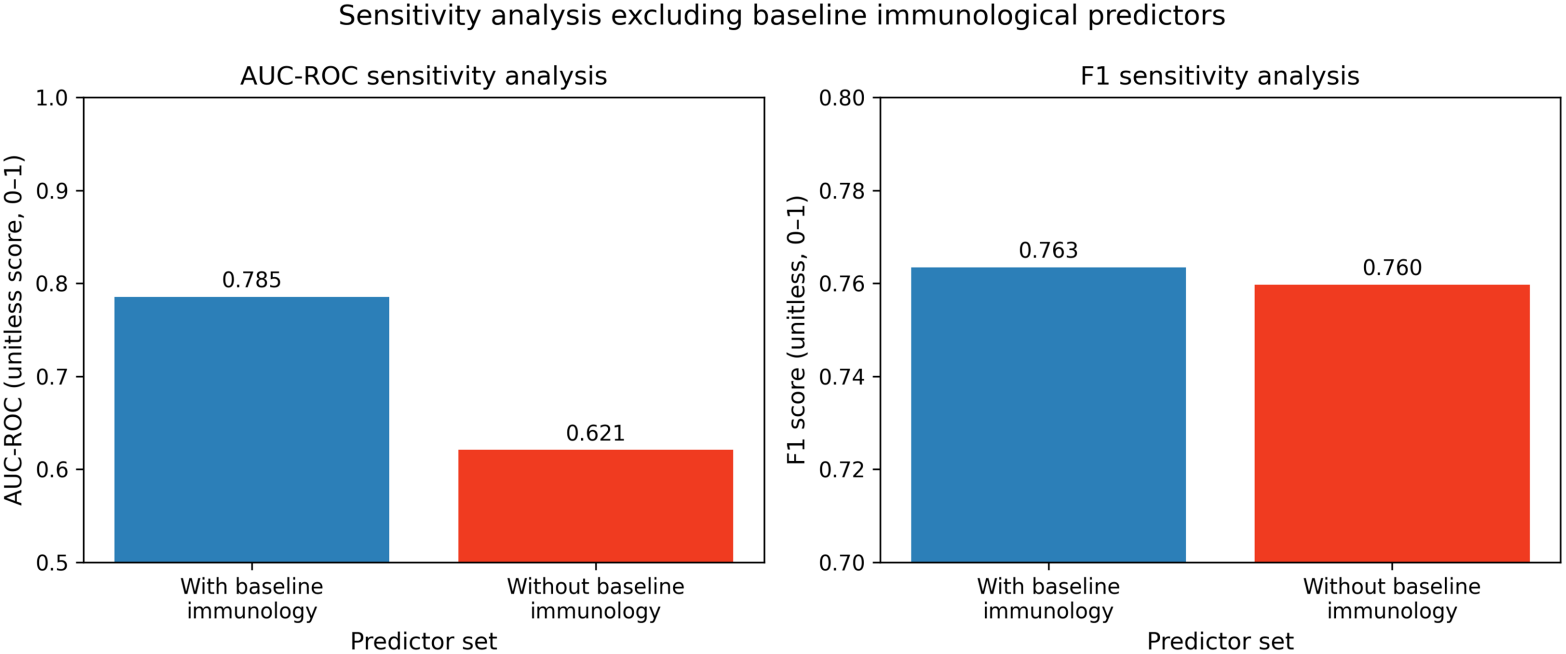
Sensitivity analysis excluding baseline immunological predictors. Left panel shows AUC-ROC and right panel shows F1 score. Excluding baseline CD4/CD8/CD4: CD8 predictors reduced AUC from 0.785 to 0.621, supporting concern about outcome-definition dependence and highlighting the need for cautious interpretation.

The stratified mean predicted risks by transmission route and baseline CD4 category (Figure 12) provide granular insights that can inform targeted interventions. For instance, individuals with transmission routes associated with higher predicted risk may benefit from tailored counseling or peer support programs (16). Importantly, the ability to generate individual-level predicted probabilities allows programs to adopt a dynamic threshold, balancing sensitivity and precision according to capacity—a hallmark of precision public health (7).

Existing machine learning applications in HIV care have predominantly focused on single outcomes—such as virological failure or loss to follow-up—with few studies integrating both delayed treatment initiation and immunological severity within a unified predictive framework, and even fewer reporting calibration or decision curve analysis. By systematically comparing multiple algorithms and demonstrating that a stacking ensemble substantially improves discrimination over individual classifiers while retaining interpretability through feature importance and stratified risk estimates, our approach addresses this gap. The clinical relevance of this work lies in its ability to provide a single, actionable risk score that simultaneously captures two high-priority programmatic outcomes: delayed ART initiation (reflecting linkage and retention gaps) and advanced immunosuppression (reflecting late diagnosis). Such a tool can be embedded into routine clinical workflows to enable earlier, more targeted interventions—for example, triggering enhanced adherence counseling, community-based support, or prioritized follow-up for individuals flagged as high-risk. Moreover, the inclusion of calibration and decision curve analysis strengthens the model’s suitability for real-world decision-making, allowing clinicians and program managers to select risk thresholds that balance sensitivity and resource constraints according to local capacity. Our exploratory component-specific analyses revealed that the composite model’s excellent performance was largely attributable to the immunological component (low baseline CD4), for which prediction was near-perfect due to the inclusion of CD4-related predictors. In contrast, prediction of delayed ART initiation alone was poor (AUC-ROC 0.560), highlighting the difficulty of capturing linkage-to-care barriers using routine demographic and clinical data. Future work should incorporate richer socio-structural variables (e.g., travel distance to clinic, stigma, mental health, social support) and consider separate modeling or two-step approaches to improve prediction of the delayed ART component.

Several limitations should be acknowledged. First, this study is based on a single retrospective cohort without external validation. While the internal validation demonstrates strong discrimination and calibration, generalisability to other populations, healthcare settings, and time periods remains unproven. Therefore, external validation using independent datasets from diverse geographic and demographic contexts is a strict prerequisite prior to any real-world implementation. Second, the definition of timely ART initiation was program-specific, which may limit comparability across settings. Furthermore, fairness metrics across protected subgroups (e.g., sex, age, ethnicity, transmission route) were not formally assessed; such evaluations are essential prior to deployment to ensure that risk stratification does not inadvertently exacerbate existing disparities. Third, the composite outcome definition includes baseline CD4 < 200 cells/μL, and CD4-related variables were among the strongest predictors. As demonstrated in the sensitivity analysis (Results section), excluding immunological predictors reduced AUC-ROC from 0.915 to 0.621, indicating substantial outcome dependence. This circularity does not invalidate the tool for its intended purpose—identifying high-risk individuals using routine data—but it does limit claims of discovering novel risk signals. Future studies using time-varying or pre-diagnosis predictors could further disentangle prediction from outcome definition. Fourth, while we present subgroup performance analyses (Table 2), formal fairness metrics (e.g., equalized odds, calibration by subgroup) were not computed. As algorithmic tools are increasingly deployed in resource-limited settings where existing disparities may be magnified, equity assessment must become a standard component of model evaluation prior to implementation. Future work should prospectively evaluate the model’s performance across demographic and social subgroups and, if disparities are detected, consider recalibration or the use of fairness-aware algorithms. Furthermore, the definition of timely ART initiation was program-specific and may have varied across facilities (e.g., different acceptable windows from diagnosis to treatment), which could introduce heterogeneity in outcome ascertainment and limit comparability across settings. Despite these limitations, the study has several strengths. It leveraged routinely collected data without imposing additional data collection burdens on clinics. The modeling approach compared six base learners alongside a stacking ensemble and included explicit evaluation of calibration and clinical utility. The analytical pipeline was designed to be modular and documented to support reproducibility. Future work should extend this approach to other outcomes, such as virological failure or loss to follow-up, and evaluate its performance in prospective implementation studies. Embedding the tool within a pilot program would enable assessment of real-world feasibility and impact. Complementary cost-effectiveness analyses would further inform threshold selection and guide decisions around scale-up, ensuring that implementation aligns with available resources and programmatic priorities.

## Conclusion

An AI-based risk stratification tool trained on routinely collected data can identify people living with HIV at high risk for delayed ART initiation or advanced immunosuppression with strong discriminative performance, good calibration, and positive net benefit across clinically relevant decision thresholds. Embedding such a tool into routine HIV programs may support precision prevention strategies and enable more efficient allocation of linkage and support services. Before wider implementation, we recommend external validation in diverse settings, formal equity assessment across demographic and clinical subgroups, and pilot integration into existing clinical and programmatic workflows.

## Data Availability

The datasets generated during the current study are not publicly available during the website review process, but are available from the corresponding author upon reasonable request. The full trial protocol is also available from the corresponding author upon request. Requests to access these datasets should be directed to xjtulhq@stu.xjtu.edu.cn.
